# Application of the behavior change wheel in supporting self-management among colorectal cancer survivors: A scoping review

**DOI:** 10.1016/j.apjon.2026.100916

**Published:** 2026-02-07

**Authors:** Siyu Lu, Qian Wu, Lizhong Wang, Xiaoli Liao, Dangheng Wei

**Affiliations:** a School of Nursing, University of South China, Hengyang, China; b Hengyang Maternal and Child Health Hospital, Hengyang, China; c Hunan Provincial People's Hospital, Changsha, China; d Key Laboratory for Arteriosclerology of Hunan Province, Hunan International Scientific and Technological Cooperation Base of Arteriosclerotic Disease, Hengyang Medical School, University of South China, Hengyang, China

**Keywords:** Colorectal cancer, Self-management, Behavior change wheel, COM-B model, Nursing interventions, Scoping review

## Abstract

**Objective:**

Self-management is a cornerstone of colorectal cancer (CRC) care; however, many interventions lack explicit grounding in behavioral theory. The Behavior Change Wheel (BCW), underpinned by the Capability–Opportunity–Motivation Behavior (COM-B) model, provides a systematic framework for designing and evaluating behavior change interventions, yet its application in CRC self-management has not been comprehensively mapped. This scoping review aimed to synthesise the existing evidence on BCW-informed studies addressing CRC self-management and screening behaviors, and to identify implications for nursing practice and future research.

**Methods:**

This scoping review was conducted in accordance with the Joanna Briggs Institute methodology and the PRISMA-ScR guideline. PubMed, Web of Science, Google Scholar, CNKI, and Wanfang were searched for studies published between April 2011 and April 2025 that applied the BCW framework (including COM-B) to CRC-related self-management or screening behaviors. Peer-reviewed articles and relevant grey literature were eligible. Study selection and data extraction were performed independently by two reviewers using a standardized form. Data were synthesised descriptively and thematically, with intervention components mapped to COM-B determinants and BCW intervention functions.

**Results:**

Ten studies met the inclusion criteria, comprising six intervention studies and four qualitative studies. These studies addressed behaviors across the CRC care continuum, including screening participation, perioperative self-care, postoperative recovery, and survivorship. Education, Enablement, and Persuasion were incorporated in all intervention studies (*n* = 6), often in combination with additional BCW functions and targeting multiple COM-B components, particularly psychological capability and reflective motivation. Qualitative findings further elucidated contextual barriers and facilitators influencing CRC-related self-management and screening behaviors. Reported outcomes included improvements in self-care ability, self-efficacy, adherence, symptom management, emotional adjustment, and screening participation.

**Conclusions:**

BCW-informed approaches show potential for strengthening key behavioral determinants and supporting patient-centered outcomes across the CRC care continuum. Integrating explicit behavioral theory into nursing interventions may enhance their conceptual rigor, transparency, and effectiveness. Further robust, theory-driven studies are warranted to refine the application of the BCW and to evaluate its impact across diverse populations and health care settings.

## Introduction

Colorectal cancer (CRC) is one of the most commonly diagnosed malignancies and is the second leading cause of cancer-related mortality worldwide. In 2022, an estimated 1.93 million new colorectal cancer cases and approximately 904,000 deaths occurred worldwide.^1^ Advances in screening, surgery, and adjuvant therapies have improved survival.^2^ However, many patients continue to experience long-term challenges across the disease trajectory, particularly when they lack the skills or support needed to manage complex treatment- and lifestyle-related behaviors.^3^^,^^4^

CRC care spans screening, active treatment, and survivorship, requiring patients to perform a wide range of self-management behaviors such as bowel preparation, ostomy and wound care, dietary and physical activity adjustments, symptom monitoring, psychological regulation, and adherence to follow-up protocols.^5^, ^6^, ^7^, ^8^, ^9^ These behaviors are dynamic, interdependent, and often difficult to sustain, particularly in the presence of emotional distress, physical limitations, or inadequate support.^10^^,^^11^ Despite the centrality of self-management to CRC outcomes, existing interventions are frequently fragmented and inconsistently implemented, with limited theoretical grounding or personalization—factors that weaken their effectiveness and long-term impact.^3^^,^^4^^,^^7^^,^^10^^,^^11^

Self-management refers to patients’ ability to manage symptoms, treatment, physical and psychosocial consequences, and lifestyle changes associated with chronic illness.^12^ In cancer care, self-management interventions have been associated with improved quality of life and other patient-reported outcomes.^13^^,^^14^ However, CRC patients often struggle to sustain required behaviors due to inadequate disease-specific knowledge, limited motivation, physical constraints, stigma associated with ostomy care, and insufficient social or health care support.^3^^,^^9^^,^^14^^,^^15^ These multifaceted and interacting barriers highlight the need for a comprehensive, theory-driven framework capable of addressing behavioral determinants at the individual, social, and environmental levels.

**Fig. 1:**
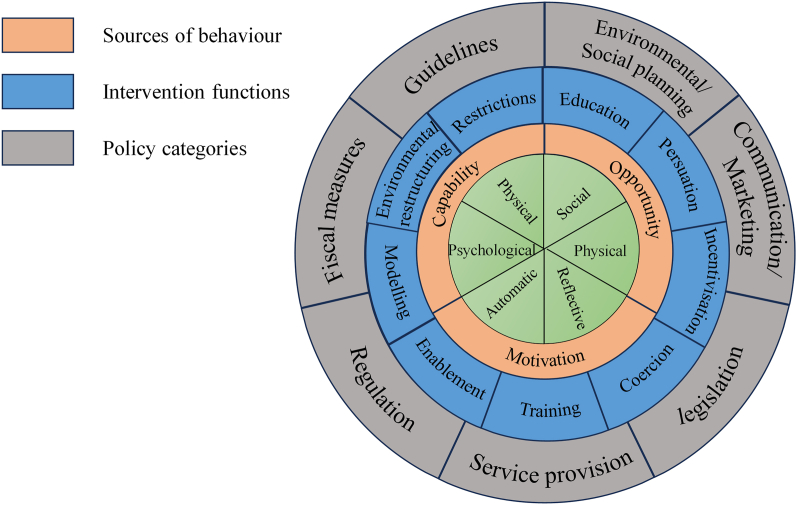
The Behavior Change Wheel. Redrawn and adapted from Michie et al.^16^ Licensed under CC BY.

**Fig. 2:**
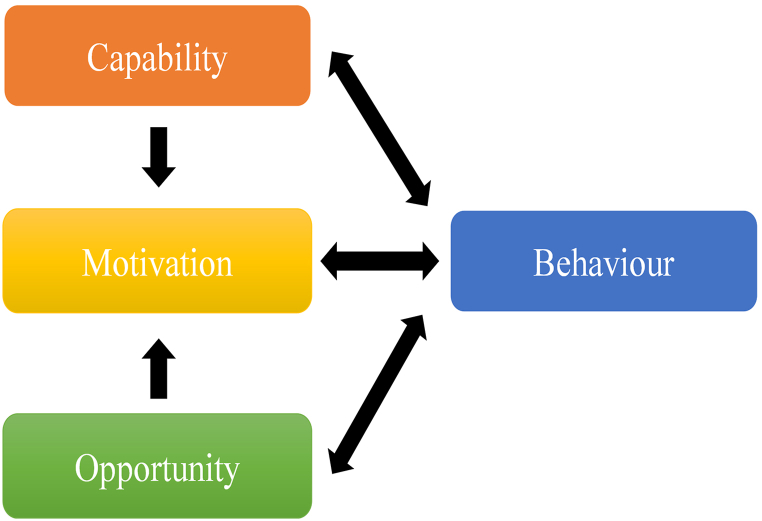
The COM-B system. Redrawn and adapted from Michie et al.^16^ Licensed under CC BY. COM-B, Capability–Opportunity–Motivation Behavior.

The Behavior Change Wheel (BCW) offers a comprehensive and theory-driven framework for designing and evaluating complex interventions by linking behavioral determinants to appropriate intervention strategies (Fig. 1). Built upon the Capability–Opportunity–Motivation Behavior (COM-B) model, the BCW conceptualizes behavior as the product of interacting psychological and physical capabilities, social and environmental opportunities, and reflective and automatic motivational processes (Fig. 2). Through its nine intervention functions—including education, persuasion, training, modelling, enablement, and environmental restructuring—the BCW provides a systematic method for selecting and tailoring strategies that address multilevel barriers to behavior change.^16^ This makes the BCW particularly suited to CRC care, where patients’ self-management challenges arise from overlapping personal, social, and contextual factors.^3^^,^^4^

Although the BCW has been increasingly applied in the management of chronic conditions such as diabetes,^17^^,^^18^ cardiovascular disease,^19^ and breast cancer,^20^ its use in CRC self-management remains limited and fragmented.^21^, ^22^, ^23^, ^24^, ^25^ To our knowledge, no review has systematically examined how BCW components have been operationalized to address the behavioral demands of CRC care. More broadly, CRC self-management interventions have often been heterogeneous and inconsistently specified, which may hinder scalability, reproducibility, and clinical impact.^4^^,^^7^^,^^11^ This gap highlights the need for a comprehensive synthesis to clarify current applications and guide the development of more rigorous, theory-driven behavioral interventions in oncology nursing.

Given these gaps, this scoping review aims to map how the BCW, including the COM-B model, has been applied to support CRC-related self-management. Specifically, it describes (1) the scope and characteristics of BCW-informed studies, including intervention and qualitative research, (2) how COM-B constructs and BCW functions have been operationalized within intervention studies, and (3) reported outcomes and implications for behavioral oncology nursing. This synthesis is intended to inform the development of more rigorous, patient-centered, and context-responsive interventions across the CRC care continuum.

## Methods

### Research questions

This scoping review followed the Joanna Briggs Institute (JBI) methodology for scoping reviews and was conducted and reported in accordance with the PRISMA extension for Scoping Reviews (PRISMA-ScR) guidelines.^26^^,^^27^ The completed PRISMA-ScR checklist is provided as a supplementary file.

In line with JBI scoping review guidance, we used the Population-Concept-Context (PCC) framework to define the review scope and eligibility criteria. Population comprised adults (≥ 18 years) across the CRC care continuum (screening, active treatment, and survivorship). Concept was the explicit application of the Behavior Change Wheel, including the COM-B model, to design, deliver, evaluate, or analytically map CRC-related self-management and screening behaviors. Context included any setting (hospital, community, home-based, or digital or remote delivery formats).

The review addressed three questions: (1) What is the scope and key characteristics of BCW-informed studies in CRC-related self-management and screening behaviors? (2) How were COM-B constructs, BCW intervention functions, and reported behavior change techniques (BCTs) operationalized? (3) What outcomes were reported, and what are the implications for behavioral oncology nursing and future intervention development?

Registration in PROSPERO was not applicable, as scoping reviews are generally excluded from this registry.

### Search strategy

A comprehensive search was conducted in five electronic databases: PubMed, Web of Science Core Collection, Google Scholar, China National Knowledge Infrastructure (CNKI), and Wanfang Data. Searches covered 23 April 2011 to 30 April 2025 and were executed on 30 April 2025.

The search strategy combined controlled vocabulary (where applicable) and free-text terms across three concepts: (1) colorectal cancer; (2) the Behavior Change Wheel (BCW) and COM-B model; and (3) self-management and related behavioral targets (e.g., screening participation and adherence, bowel preparation, ostomy care, symptom monitoring, lifestyle behaviors, and follow-up). A representative PubMed search strategy is provided in Appendix 1.

Search results from each database were recorded and exported for screening. Complete database-specific syntaxes, fields, limits, search dates, and yields are provided in Supplementary Table S1.

### Inclusion criteria

Studies were eligible for inclusion if they met the following criteria:

Concept: explicitly applied the BCW or COM-B model to guide the development, implementation, or evaluation of self-management–related behaviors in CRC-related populations.

Population: included adults (≥ 18 years) across the CRC care continuum, including those diagnosed with CRC or undergoing CRC-related care, as well as adults eligible for CRC screening. Studies with mixed cancer samples were eligible if CRC participants comprised ≥ 10% of the sample, and the CRC subgroup was clearly reported.

Self-management definition: addressed behavioral processes supporting long-term health management, such as symptom monitoring, medication adherence, dietary management, lifestyle modification, or other relevant self-care behaviors.

Time frame: published in or after 2011, consistent with the introduction of the BCW framework.

Language: published in English or Chinese.

Given the emerging nature of this field, both peer-reviewed literature and eligible gray literature (e.g., academic theses) were included to increase comprehensiveness and reduce publication bias.

### Exclusion criteria

Studies were excluded if they met any of the following criteria:

Publication type: conference abstracts, editorials, commentary papers, news articles, or other non-scholarly sources. Academic theses were retained if they met methodological standards.

Conceptual relevance: did not address CRC-related self-management or screening behaviors, nor their associated behavioral determinants, or mentioned the BCW/COM-B model without applying it to intervention design or analysis.

Population: included mixed cancer samples in which CRC patients represented < 10% of participants, or exclusively included non-CRC populations.

Availability: lacked full-text access or did not provide adequate methodological information to support appraisal and data extraction.

### Data extraction and synthesis of results

Two reviewers independently screened all records and assessed eligibility based on the predefined criteria. Reference management and duplicate removal were performed using Zotero 7.0.

Data were extracted using a standardized Microsoft Excel form, capturing study characteristics (authors, year, country, design, sample), target behaviors, COM-B determinants, BCW intervention functions, BCTs, intervention duration, and outcome direction. Discrepancies were resolved through discussion or, when necessary, consultation with a third reviewer.

Extracted information was organized into structured summary tables (Table 1, Table 2, Table 3; Supplementary Table S2) to provide a transparent overview of quantitative and qualitative findings. Quality appraisal was conducted using the Cochrane risk-of-bias tool for quantitative studies^28^ and the JBI Critical Appraisal Checklist for qualitative studies.^29^ A descriptive and thematic synthesis approach was used. Themes were developed by grouping extracted findings according to COM-B domains and BCW functions and were refined through reviewer discussion.

**Table 1:** Characteristics of included randomized and quasi-experimental studies.

Author, Year (Country)	Design & Setting	Sample	Intervention	Comparator	Duration	Primary outcomes
Yan J et al., 2022 (China)	RCT; surgical ward + mobile platform	CRC stoma patients (*n* = 96)	Intervention: COM-B self-care program (mobile delivery). Target behavior: daily stoma self-care. BCW functions: Edu + Train + Enb	Routine stoma education	3 months post-discharge	ESCA ↑; Stoma-QOL ↑; GSES ↑ (*P* < 0.05). Effect sizes not reported.
Wang Y & Zhu GL, 2020 (China)	RCT; colorectal surgical ward	Post-operative CRC stoma patients (*n* = 94)	Intervention: BCW nursing program (structured education, assessment, feedback). Target behavior: perioperative self-management. BCW functions: Edu + Pers + Enb	Routine postoperative care	1 month	SMQ-EP ↑; KAP ↑ (*P* < 0.05). Effect sizes not reported.
Kerrison RS et al., 2018 (UK)	RCT; community bowel screening	Adults aged 55 y (*n* = 1383; 3-arm trial)	Intervention: two self-referral reminders + BCW leaflet. Target behavior: flexible sigmoidoscopy screening uptake. BCW functions: Pers + Edu + Enb + Mod + EnvR	Standard leaflet / no reminder	Reminders over 12–24 months; 12-week follow-up	Screening uptake ↑ (*P* < 0.001). Effect sizes not reported.
Guo JY, 2019 (China)	RCT; tertiary stoma clinic	Permanent CRC stoma patients (*n* = 80)	Intervention: BCW stoma-care education (6 sessions + demo + WeChat follow-up). Target behavior: daily stoma self-care. BCW functions: Edu + Train + Pers + Enb + Mod + EnvR	Routine stoma education	3 months post-discharge	ESCA ↑; HPLP-II ↑ (*P* < 0.05). Effect sizes not reported.
Ze Y, 2023 (China)	RCT; community CRC screening	High-risk population (*n* = 200)	Intervention: BCW comprehensive program (education, calls, MI, follow-up). Target behavior: colonoscopy attendance. BCW functions: Edu + Pers + Enb + Train + Mod + EnvR	Standard reminder	3 months	HBMQ ↑; colonoscopy uptake ↑ (*P* < 0.05). Effect sizes not reported.
Zhu T., 2023 (China)	Quasi-experimental; outpatient endoscopy centre	CRC polypectomy patients (*n* = 201)	Intervention: BCW self-management program (education, persuasion, training, environmental restructuring; handbook + video). Target behavior: bowel preparation & perioperative self-management. BCW functions: Edu + Train + Pers + EnvR	Routine nursing care	1 month	BBPS ↑; AHSMSRS ↑; MMAS-8 ↑; HADS ↓ (*P* < 0.05). Effect sizes not reported.

CRC, colorectal cancer; Edu, education; Pers, persuasion; Train, training; Enb, enablement; Mod, modelling; EnvR, environmental restructuring; COM-B, Capability–Opportunity–Motivation Behavior; BCW, Behavior Change Wheel; ESCA, Exercise of Self-Care Agency Scale; Stoma-QOL, Stoma Quality of Life Scale; GSES, General Self-Efficacy Scale; SMQ-EP, Stoma Management Questionnaire for Enterostomy Patients; KAP, Knowledge–Attitude–Practice; HPLP-II, Health-Promoting Lifestyle Profile II; HBMQ, Health Belief Model Questionnaire; BBPS, Boston Bowel Preparation Scale; AHSMSRS, Adult Health Self-Management Skills Rating Scale; MMAS-8, 8-item Morisky Medication Adherence Scale; HADS, Hospital Anxiety and Depression Scale; RCT, randomized controlled trial. ↑, improvement/increase; ↓, reduction.

**Table 2:** Characteristics of included qualitative studies (mapped to COM-B).

Author, Year (Country)	Design & Setting	Sample	Data Collection	Key themes	Mapped COM-B components
Christie-de Jong et al., 2022 (UK)	Qualitative; faith-based community setting (Scotland)	Muslim women (*n* = 20; 18–65 y)	FGD + semi-structured interviews	Faith-driven motivation; supportive community norms; embarrassment and cultural barriers; mistrust of screening materials	M-Ref; O-Soc
Gadd et al., 2024 (Australia)	Qualitative; rural CRC screening outreach (Tasmania)	Adults eligible for CRC screening (*n* = 15; 50–75 y)	Semi-structured interviews	Limited awareness; literacy barriers; stigma/taboo; social support needs; privacy and logistical concerns	C-Psych; O-Soc; O-Phys
Kotte et al., 2024 (Sweden)	Qualitative; remote supervised exercise program (online)	Cancer survivors (*n* = 24; 30–65 y; mixed tumour types; CRC subgroup = 13.6%)	Semi-structured interviews + short survey	Perceived exercise benefits; fluctuating motivation; peer support; desire for structured guidance; competing demands	M-Ref; M-Aut; C-Phys; O-Soc; O-Phys
Wang XY, Zhu XP & Wu Q, 2023 (China)	Qualitative; hospital + community prehabilitation	Older CRC patients (*n* = 30; 65–80 y)	Semi-structured interviews	Motivation to recover; need for professional guidance; reliance on family support; physical limitations	C-Phys; C-Psych; O-Soc; M-Ref

CRC, colorectal cancer; COM-B, Capability–Opportunity–Motivation Behavior; C-Psych, psychological capability; C-Phys, physical capability; O-Soc, social opportunity; O-Phys, physical opportunity; M-Ref, reflective motivation; M-Aut, automatic motivation; FGD, focus group discussion; y, years.

**Table 3:** Intervention mapping for included studies.

Author, Year (Country)	Barriers/Facilitators	Mapped COM-B components	Target behavior	BCW intervention functions	BCTs (reported/inferred∗)	Outcome direction	Mechanistic notes
Yan J et al., 2022 (China)	Knowledge deficit; limited skills; anxiety; need for follow-up; peer support (+)	C-Psych; C-Phys; M-Ref; M-Aut; O-Soc	Daily stoma self-care	Edu; Train; Pers; Enb; Mod	Instruction; demonstration; feedback; social support∗; action planning∗	Self-care ability ↑	Behavior change likely driven by capability ↑ and ongoing follow-up support.
Wang Y & Zhu GL, 2020 (China)	Poor knowledge; low confidence; post-op anxiety; dependence on nursing support	C-Psych; C-Phys; M-Ref; M-Aut; O-Soc	Perioperative stoma self-management	Edu; Train; Enb; Pers; EnvR	Instruction; demonstration; feedback; action planning∗	SMA ↑, KAP ↑	Improved capability + perioperative guidance strengthened self-management behavior.
Kerrison RS et al., 2018 (UK)	Fear/embarrassment; low perceived need; logistical barriers; benefit framing (+); reminders (+)	C-Psych; M-Ref; M-Aut; O-Soc	Self-referral for FS CRC screening	Edu; Pers; Enb; Mod; EnvR	Information about consequences; instruction; prompts/cues; action planning∗; pros & cons∗	FS uptake ↑	Motivation ↑ + timely prompts enhanced screening uptake.
Guo JY, 2019 (China)	Knowledge/skill deficit; low confidence; reliance on nurses/family; anxiety about appearance/odour; family involvement (+); structured practice (+); WeChat follow-up (+)	C-Psych; C-Phys; M-Ref; M-Aut; O-Soc	Daily stoma self-care	Edu; Pers; Train; Mod; Enb; EnvR	Instruction; demonstration; feedback; information about consequences; social support; prompts/cues; action planning∗; graded tasks∗	Self-care ability ↑, complications ↓	Enhanced capability + motivation + social support improved self-care behavior.
Ze Y, 2023 (China)	Fear/embarrassment; low perceived risk; misconceptions; logistical barriers; weak support; clear information (+); fast-track appointment (+); multi-modal follow-up (+)	C-Psych; C-Phys; M-Ref; M-Aut; O-Soc	Colonoscopy attendance	Edu; Train; Pers; Enb; Mod; EnvR	Information about consequences; instruction; demonstration; behavioral practice; social support; prompts/cues; action planning∗; problem solving∗	HBMQ ↑; colonoscopy ↑ uptake; anxiety ↓	Screening behavior improved via ↑ capability, reminders, and structural/social support.
Zhu T, 2023 (China)	Insufficient perioperative self-management capability; limited resources/opportunity; low motivation; education (+); family support (+); risk awareness (+)	C-Psych; C-Phys; O-Soc; O-Phys; M-Aut; M-Ref	Colonoscopy follow-up adherence	Edu; Train; Pers; Enb; Mod; EnvR	Information about consequences; instruction; demonstration; feedback; social support; prompts/cues; action planning; graded tasks∗	Adherence ↑, complications ↓	Behavior change driven by improved capability, motivation, and supportive environment.
Christie-de-Jong et al., 2022 (UK)	Low CRC screening knowledge; embarrassment; cultural concerns; low perceived risk; mistrust; faith-based messaging (+); trusted community voices (+); culturally adapted materials (+)	C-Psych; M-Ref; M-Aut; O-Soc	CRC screening uptake intention	Edu; Pers; Mod; Enb	Information about consequences; credible source; social support; reframing; reduced negative emotions; action planning∗	Screening intention ↑	Capability ↑ + trust ↑ + culturally aligned messaging ↑ strengthened motivation.
Gadd et al., 2024 (Australia)	Low awareness; stigma/taboo; limited support; literacy barriers; privacy concerns; GP encouragement (+); family/peer support (+); simple kit materials (+)	C-Psych; C-Phys; M-Ref; M-Aut; O-Soc; O-Phys	Bowel cancer screening (iFOBT completion)	Edu; Pers; Enb; Train; Mod; EnvR	Information about consequences; prompts/cues; social support (practical/emotional); restructuring the environment∗; action planning∗; demonstration∗	Screening intention/uptake ↑	Capability ↑ + reduced stigma + simple kit materials + social/GP support were associated with increased screening uptake
Kotte et al., 2024 (Sweden)	Limited exercise capability; low confidence; physical challenges; time constraints; variable motivation; remote access (+); trainer support (+); peer support (+)	C-Psych; C-Phys; M-Ref; M-Aut; O-Phys; O-Soc	Post-treatment exercise participation	Edu; Train; Pers; Enb; Mod; EnvR	Instruction; demonstration; feedback; action planning∗; goal setting∗; social support; environment restructuring∗	Engagement ↑	Digital accessibility + enhanced capability + trainer/peer support improved exercise behavior.
Wang XY, Zhu XP & Wu Q, 2023 (China)	Low CRC-related exercise knowledge; weak self-efficacy; fear of complications; lack of tailored guidance; time constraints; professional support (+); family encouragement (+)	C-Psych; C-Phys; M-Ref; M-Aut; O-Soc	Preoperative exercise adherence	Edu; Train; Pers; Enb; Mod	Instruction; demonstration; feedback; action planning∗; social support∗	Prehabilitation adherence ↑	Improved capability + tailored advice + family/professional support increased adherence.

COM-B, Capability–Opportunity–Motivation Behavior; C-Psych, psychological capability; C-Phys, physical capability; O-Soc, social opportunity; O-Phys, physical opportunity; M-Ref, reflective motivation; M-Aut, automatic motivation; BCW, Behavior Change Wheel; Edu, education; Pers, persuasion; Train, training; Enb, enablement; Mod, modelling; EnvR, environmental restructuring; BCTs, Behavior Change Techniques; FS, flexible sigmoidoscopy; SMA, self-management ability; KAP, knowledge, attitudes and practices; HBMQ, Health Belief Model Questionnaire; iFOBT, immunochemical fecal occult blood test. (+), facilitator; ↑, increase; ↓, decrease: ∗, inferred.

**Fig. 3:**
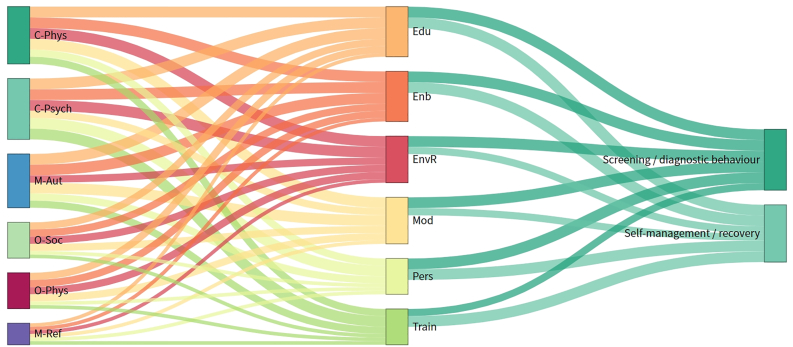
Sankey diagram illustrating linkages between COM-B components, BCW intervention functions, and behavioral outcomes. Flow thickness indicates the frequency of each linkage across included studies. Overall, opportunity-related pathways—particularly physical opportunity—were less frequently represented than capability and motivation pathways. An interactive HTML version is available in the Supplementary Materials. COM-B, Capability–Opportunity–Motivation Behavior.

To enhance interpretability, a Sankey diagram (Fig. 3) was constructed to visualize the relationships between COM-B components, BCW functions, and outcome categories, with flow thickness representing frequency across studies.

Ethical approval was not required because the review used data from publicly available published sources.

## Results

### Study selection

A total of 102 records were identified across database and gray-literature sources. After removing 13 duplicates, 89 unique records underwent title and abstract screening. Four records—one conference abstract and three study protocols—were excluded because they did not contain complete empirical data suitable for evidence synthesis. The remaining 85 full texts were assessed for eligibility, of which 10 studies met all inclusion criteria and were included in the final synthesis.

**Fig. 4:**
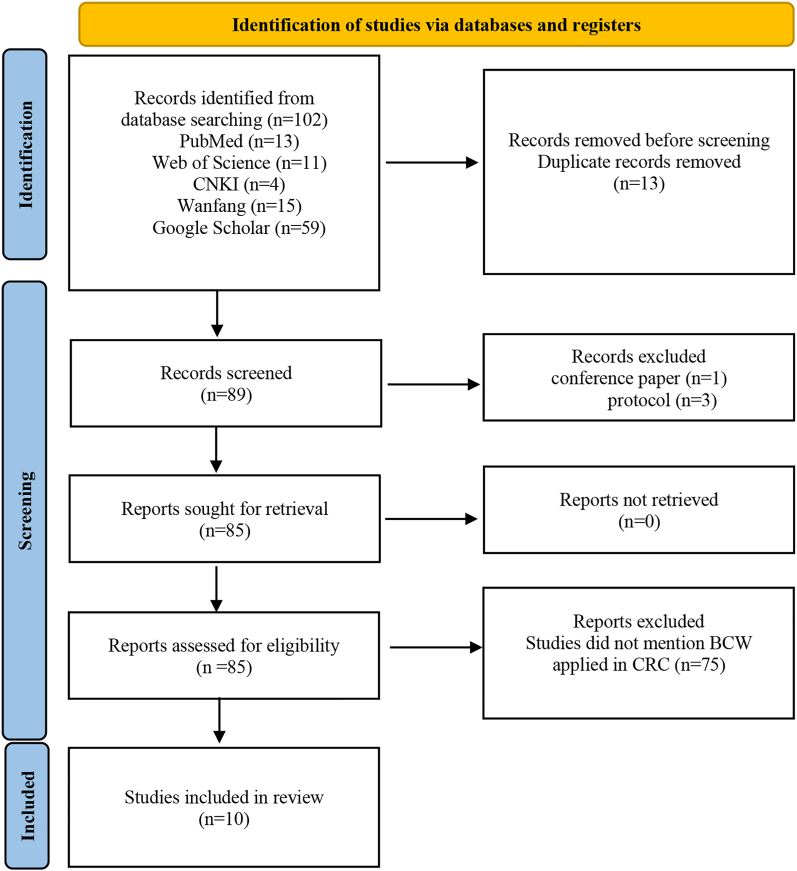
PRISMA flow diagram of study selection. Adapted from Page MJ et al.^34^ CNKI, Chinese National Knowledge Infrastructure; Wanfang, Wanfang Data. PRISMA, Preferred Reporting Items for Systematic Reviews and Meta-Analyses.

Gray literature (three master's theses) was retained because it met the predefined methodological and topical relevance criteria and contributed additional empirical evidence not captured in peer-reviewed sources. The full study selection process is presented in the PRISMA-ScR flow diagram (Fig. 4).

### Study characteristics

The final sample included six quantitative studies (three peer-reviewed RCTs^6^^,^^21^^,^^22^ and three master's theses reporting RCT or quasi-experimental designs^9^^,^^23^^,^^25^) and four qualitative studies exploring patient or public perspectives.^14^^,^^30^, ^31^, ^32^ The studies were published between 2018 and 2024 and were conducted in China (*n* = 6), the United Kingdom (*n* = 2), Australia (*n* = 1), and Sweden (*n* = 1).

Across studies, target populations included CRC survivors with stoma, patients undergoing polypectomy, high-risk adults eligible for colonoscopy, and community-dwelling adults eligible for population-based screening programs. Behavioral targets varied accordingly, ranging from stoma self-care and postoperative self-management to screening uptake and adherence to exercise or prehabilitation programs.

Gray-literature sources (i.e., master's theses) met predefined methodological and topical relevance criteria and contributed additional data not available in the peer-reviewed literature. Detailed characteristics of the quantitative and qualitative studies are presented in Table 1, Table 2, respectively.

### Intervention characteristics

#### Quantitative interventions

Across the six quantitative studies, interventions were explicitly developed using the COM-B model and BCW, with most targeting multiple behavioral determinants simultaneously. Psychological capability (knowledge, skill acquisition) and reflective motivation were the most consistently addressed COM-B components, followed by physical capability and social opportunity. Physical opportunity was least commonly incorporated.

Interventions typically combined several BCW functions—most frequently Education, Training, Persuasion, Enablement, and Environmental Restructuring—often delivered in multi-component formats. Common BCTs included instruction, demonstration, individualized feedback, social support, prompts/cues, and action planning, as detailed in Table 3 and Supplementary Table S2.

Intervention duration varied substantially across studies, ranging from brief multi-week perioperative programs to extended reminder-based programs lasting up to 12–24 months, with most lasting 1–3 months following discharge. Delivery formats included face-to-face sessions, printed or video-based materials, digital health platforms (*e.g.*, WeChat), and structured follow-up programs.

#### Qualitative findings elucidating COM-B determinants

Although the four qualitative studies did not involve behavioral interventions, their findings directly mapped onto COM-B constructs and provided contextual explanations for how BCW-based interventions may exert their effects. Participants consistently emphasized psychological capability (knowledge, clarity of instructions), social opportunity (family, peer, or faith-based support), and reflective motivation (confidence, perceived value) as central drivers of behavior change. Barriers included limited access to resources, time constraints, and low perceived control. These themes align with BCW functions such as Education, Training, and Enablement, reinforcing the COM-B determinants targeted in quantitative interventions.

### Quality appraisal

#### Quantitative studies

All six quantitative studies were rated as having an overall high risk of bias according to the Cochrane Risk of Bias tool. The domains most frequently raising concern were blinding of participants and personnel (5/6 studies rated high, 1/6 unclear) and allocation concealment (4/6 unclear, 1/6 high, 1/6 low). Blinding of outcome assessment was judged as low risk in three studies and unclear in the remaining three. In contrast, most studies were rated as low risk for incomplete outcome data and selective reporting (5/6 low, 1/6 unclear in each domain), suggesting that attrition and outcome reporting were generally well managed. These patterns reflect common structural challenges in behavioral and nursing intervention trials, where full blinding and detailed reporting of allocation procedures are often difficult to achieve in practice.

#### Qualitative studies

The four qualitative studies demonstrated moderate to high methodological quality, with JBI checklist scores ranging from 8/10 to 10/10. Overall, there was good congruity between the stated philosophical perspective, methodology, research questions, data collection, analysis, and interpretation. The most frequent limitations were insufficient reporting of researcher positionality and limited discussion of the researcher's influence on the research process. Despite these gaps in reflexivity, all studies clearly represented participants' voices and reported ethical approval.

Taken together, the included studies provide acceptable methodological support for a scoping review. High overall risk of bias in the quantitative studies is mainly driven by challenges in blinding and allocation reporting rather than by fundamental flaws in study design, while the qualitative evidence is generally robust with minor reflexivity-related limitations. Detailed appraisal results are presented in Supplementary Table S3 and Table S4.

### Synthesis across studies

Across the ten included studies, convergent patterns emerged in behavioral determinants, BCW intervention functions, and behavioral outcomes, despite heterogeneity in study design, populations, and behavioral targets.

#### COM-B determinants and behavioral targets

Most studies targeted multiple COM-B components simultaneously, with psychological capability (knowledge, understanding, self-management skills), reflective motivation (beliefs, perceived benefits, confidence), and social opportunity (family, peer, and professional support) appearing most frequently. Physical capability was commonly addressed in self-management or exercise-related interventions, whereas physical opportunity was least represented, typically through modifications to access or resources.

Behavioral targets fell into two primary categories: (1) treatment-related self-management behaviors (*e.g.*, stoma care, perioperative self-management, exercise adherence), and (2) CRC screening behaviors, including uptake of endoscopic procedures (sigmoidoscopy or colonoscopy), completion of stool-based tests (iFOBT, immunochemical fecal occult blood test), and screening intentions.

Together, these illustrate that COM-B–informed approaches were applied across both treatment and prevention contexts.

#### BCW functions and BCT patterns

All quantitative interventions used multiple BCW functions, most frequently Education, Persuasion, and Enablement, often accompanied by Training and—less consistently—Modelling or Environmental Restructuring. Frequently observed BCTs included instruction, demonstration, personalized feedback, information about consequences, social support, prompts/cues, and action planning.

The most recurrent functional combination—Education + Enablement (with or without Training/Persuasion)—was associated with positive changes across both self-management and screening behaviors.

Qualitative findings, although not coded into BCW functions, aligned closely with these categories. Participants’ expressed needs for clear guidance, trusted information, supportive communication, and culturally congruent messaging map onto Education, Persuasion, and Enablement, helping to explain why these functions may be particularly influential in CRC-related behavior change.

#### Quantitative studies: outcomes and effect directions

All six quantitative studies reported directional improvements in their targeted outcomes. For self-management interventions, outcomes such as self-care ability, self-efficacy, lifestyle behaviors, knowledge-attitude-practice, and psychological wellbeing improved. Screening-focused interventions reported higher procedure uptake, improved bowel preparation, and enhanced screening beliefs or medication adherence.

However, substantial heterogeneity was observed across measurement tools, intervention intensity, and follow-up periods (ranging from single-session perioperative interventions to multi-month or year-long programs). Importantly, no study reported standardized effect sizes, limiting comparability of intervention magnitude and precluding meta-analysis. The current review therefore synthesizes effects narratively, focusing on direction rather than magnitude, as summarized in Supplementary Table S2.

#### Qualitative studies: themes mapped to COM-B

The four qualitative studies contributed explanatory depth by illustrating how behavioral determinants shape engagement with CRC self-management and screening. Themes mapped onto psychological capability (information needs, procedural understanding), physical capability (fatigue, physical limitations), social opportunity (family, peer, faith, and clinical support), and reflective and automatic motivation (beliefs, fears, emotional responses, habit).

Enablers included clear, trustworthy information; culturally or faith-aligned communication; and structured, empathetic support. Barriers included stigma, embarrassment, time constraints, and limited access to services. These findings reinforce and elaborate the COM-B determinants (barriers and facilitators) targeted in COM-B–based interventions.

#### Cross-study convergence and heterogeneity

Overall, quantitative improvements and qualitative themes showed strong convergence: interventions that enhanced psychological capability, reflective motivation, and social opportunity—operationalized through Education, Training, Persuasion, and Enablement—were frequently linked with positive behavioral outcomes in the included studies.

A visual mapping of COM-B components, BCW functions, and behavioral outcomes (Fig. 3), where flow thickness indicates linkage frequency, further supported these patterns. The most frequently observed links connected psychological capability with Enablement, automatic motivation with Training, and social opportunity with Modelling. In the BCW-to-outcome stage, Training was most often associated with self-management and recovery outcomes, whereas Environmental Restructuring was more commonly linked with screening and diagnostic behaviors. These visual patterns reinforce the predominance of multi-component strategies focusing on capability and motivation, while opportunity-related determinants, particularly physical opportunity, were comparatively less addressed.

Nonetheless, heterogeneity in study designs, populations, settings, intervention duration, and outcome measures—the absence of standardized effect sizes in particular—necessitated a narrative and thematic synthesis rather than quantitative pooling. Despite these variations, the consistency of patterns across diverse contexts strengthens the interpretability of findings.

## Discussion

### Summary of main findings

This scoping review synthesized ten studies that applied the COM-B model and Behavior Change Wheel to CRC-related self-management and screening behaviors, including six intervention studies and four qualitative studies that elucidated COM-B–related barriers and facilitators. Across the six intervention studies, the interventions most consistently targeted psychological capability, reflective motivation, and social opportunity. These were addressed using BCW functions such as Education, Training, Persuasion, and Enablement, which were operationalized through behavior change techniques including instruction, demonstration, feedback, social support, and action planning.

Quantitative studies reported directional improvements in outcomes such as self-care ability, self-efficacy, symptom management, and screening participation, whereas qualitative studies provided explanatory insights into COM-B determinants by highlighting information needs, cultural alignment, supportive communication, and confidence-building processes. These convergent findings suggest that COM-B and BCW offer a theoretically coherent and pragmatically flexible framework for CRC behavior change interventions.

Nonetheless, substantial heterogeneity in intervention content, duration, delivery format, and outcome measurement—along with limited reporting of effect sizes—restricted comparability across studies and precluded meta-analysis. These limitations underscore the need for more standardized behavioral outcomes, transparent reporting, and stronger theory-driven intervention design in future BCW-informed CRC research.

### Contributions of COM-B and BCW to CRC behavioral interventions

Across the included intervention studies, COM-B and the BCW contributed to intervention design by providing a structured pathway from behavioral diagnosis to intervention selection. Across prehabilitation, postoperative recovery, survivorship, and community-based screening contexts, COM-B analyses most commonly identified gaps in psychological capability, reflective motivation, and social opportunity, which in turn guided the use of BCW functions such as Education, Training, Persuasion, and Enablement. These functions were operationalized through BCTs including instruction, demonstration, feedback, goal setting, and social support.

BCW-informed interventions demonstrated flexibility across clinical phases. Perioperative and postoperative programs enhanced postoperative recovery, self-care ability, and confidence through capability-building strategies, while survivorship interventions targeted lifestyle adjustment and psychological adaptation.^8^^,^^13^ BCW's adaptability was also evident in culturally tailored community interventions, such as the program among Scottish Muslim women, which incorporated faith-aligned messaging and community-led engagement to address motivation and social opportunity barriers.^30^ Technology-supported formats—mobile applications, online groups, and remote follow-up—further extended reach and acceptability, particularly for patients with limited access to in-person services.^7^^,^^24^^,^^33^

By linking COM-B determinants to specific BCW functions and techniques, these studies illustrate the framework's practical utility for designing theoretically coherent interventions tailored to population needs and clinical timing.

### Under-addressed opportunity components and implications for sustainability

Across the included studies, opportunity-related determinants—particularly physical and social opportunity—were addressed far less frequently than capability and motivation. Most interventions focused on enhancing knowledge, skills, and self-efficacy, but few systematically modified environmental or social conditions that shape CRC-related behaviors. This imbalance reflects a broader pattern in behavioral intervention research, wherein individual-level strategies are prioritized even when contextual constraints remain substantial.

Physical opportunity barriers such as limited access to screening services, time constraints, transportation challenges, and fragmented follow-up pathways were seldom explicitly targeted. Similarly, social opportunity—although recognized in qualitative findings as a key facilitator of behavior change—was inconsistently operationalized in intervention design. Peer support, family involvement, and community engagement were mentioned but rarely embedded as structured components, with the exception of culturally tailored community-led interventions.

The underuse of opportunity-oriented strategies has important implications for intervention sustainability. Without changes to social norms, care systems, or environmental enablers, improvements in knowledge or motivation may not translate into durable behavioral change. For example, enhanced self-efficacy or motivation may not be sufficient if participants continue to experience logistical barriers to attending screening or lack support in maintaining lifestyle changes.

Integrating BCW functions such as Environmental Restructuring, Modelling, and Enablement, alongside multi-level strategies (*e.g.*, streamlined referral systems, culturally grounded community advocacy, institutional reminders, or family-inclusive care), may help create supportive environments that reinforce and sustain behavioral gains. Future CRC interventions should therefore adopt a more balanced COM-B approach that systematically targets capability, motivation, and opportunity to promote enduring behavioral change.

### Implications for nursing practice

The findings of this review provide several practice-level implications for nurses delivering CRC-related behavioral interventions. First, the consistent identification of capability- and motivation-related barriers underscores the importance of conducting a COM-B–informed behavioral diagnosis as an initial step in clinical assessment. Nurses can apply this structured diagnostic process to identify gaps in knowledge, procedural skills, emotional readiness, and social support, ensuring that interventions are tailored to the specific behavioral determinants experienced by each patient.

Second, the frequent use of BCW functions such as Education, Training, Persuasion, and Enablement highlights their practical relevance to routine nursing care. These functions can be operationalized through BCTs such as demonstration, action planning, problem-solving, feedback, reinforcement, and mobilization of social support—strategies that can be delivered during prehabilitation consultations, postoperative follow-up, survivorship care planning, and screening outreach. Embedding these components into standardized care pathways may improve consistency, scalability, and quality of behavioral support.

Third, the qualitative findings emphasise the value of supportive communication—including culturally attuned messaging, empathy, role modelling, and collaborative decision-making—in strengthening reflective motivation and patient trust.^3^^,^^33^ Nurses occupy a uniquely influential position in shaping social opportunity through family engagement, peer support facilitation, and culturally sensitive health communication, especially in communities with distinctive beliefs or health literacy needs.^30^^,^^31^

Finally, the limited use of opportunity-enhancing strategies in existing interventions indicates a need for nurses to advocate for system-level enablers. These may include streamlined referral pathways, reminder systems, coordinated follow-up mechanisms, and partnerships with community organizations to reduce environmental and logistical barriers to behavior change. Such multi-level efforts may reinforce the sustainability of behavioral gains and support long-term adherence across the CRC care continuum.

### Implications for future research

Future CRC behavioral research should strengthen the theoretical integrity and methodological rigor of BCW-guided interventions. First, studies should adopt a complete COM-B behavioral diagnosis as the foundation for intervention development, explicitly linking identified behavioral determinants to BCW intervention functions and corresponding BCTs. Such transparent mapping would improve intervention fidelity, enhance replicability, and facilitate meaningful comparison across studies.

Second, the systematic use of the Behavior Change Technique Taxonomy (BCTTv1) is needed to classify, standardise, and report intervention components. Several included studies referenced BCW superficially without specifying the BCTs used, leading to ambiguity regarding active ingredients. Future trials should document BCT selection, dosage, delivery mode, and fidelity using established reporting frameworks such as TIDieR or CONSORT extensions for behavioral interventions.

Third, research should incorporate mechanistic and process-oriented evaluations to clarify how COM-B components change over time and through which pathways interventions exert their effects. Mediation and moderation analyses, alongside qualitative process evaluations, can help identify differential response patterns, contextual contingencies, and essential components that drive behavioral change or limit impact.

Fourth, greater standardisation of outcome measures is needed. The current evidence base is characterised by substantial heterogeneity in behavioral, psychosocial, and clinical indicators, with limited reporting of effect sizes or long-term follow-up. Developing a core outcome set for CRC behavioral interventions—including measures of capability, motivation, opportunity, adherence, and patient-reported outcomes—would enhance comparability and cumulative knowledge building.

Finally, future studies should adopt multi-level and real-world implementation approaches, addressing structural and social opportunity barriers through system-level strategies, community partnerships, and culturally tailored models. Well-powered, multi-site, and longitudinal trials are essential to test scalability, sustainability, and external validity, particularly in diverse settings where CRC risk, resources, and cultural norms vary.

### Strengths and limitations

This review has several strengths. It is the first to provide a theory-informed synthesis of COM-B and BCW applications in colorectal cancer behavior change, integrating quantitative, qualitative, and gray literature sources to capture emerging evidence across the CRC care continuum. The use of a structured, theory-driven analytical framework enabled a deeper examination of behavioral determinants, intervention functions, and BCTs, extending beyond descriptive categorization to highlight cross-study patterns and conceptual gaps. The inclusion of gray literature—while introducing variability in reporting quality—enhanced comprehensiveness and reduced publication bias, offering insight into early-stage intervention development and culturally specific applications.

Several limitations should also be acknowledged. Consistent with scoping review methodology, this review did not conduct meta-analysis or formal effectiveness synthesis, and the heterogeneity in intervention design, outcome measures, and follow-up periods limited direct comparison across studies. Reporting quality varied, particularly among theses and early-phase trials, leading to incomplete information on BCT specification, intervention fidelity, and effect sizes. Although quality appraisal was undertaken to support interpretive depth, the findings should be interpreted cautiously due to variability in methodological rigor, small sample sizes, and context-specific features. To obtain a comprehensive understanding of how the BCW has been applied to CRC-related self-management and screening behaviors, we searched both English- and Chinese-language databases from the outset. This strategy reduced language and indexing bias and allowed us to capture evidence that may be missed by English-only searches. Consequently, included studies were geographically concentrated, with more than half conducted in China (6/10), which may limit transferability to other health systems and cultural contexts. This concentration may also partly reflect the substantial CRC burden in several Asian countries and the growing volume of CRC-related research from this region.^1^ However, evidence from other health systems remains limited, highlighting the need for cross-regional validation and culturally adaptable intervention models. Nevertheless, qualitative findings highlighted culturally and socially shaped influences (*e.g.*, stigma/embarrassment and reliance on family support), reinforcing the importance of context-sensitive intervention design and cross-regional evaluation.

## Conclusions

This scoping review provides the first theory-informed synthesis of COM-B and Behavior Change Wheel applications in colorectal cancer–related self-management and screening behaviors. Despite a limited and heterogeneous evidence base, the available evidence consistently demonstrates that BCW-guided approaches can strengthen key behavioral determinants—particularly capability and motivation—and show potential for improving self-management, adherence, and screening engagement across the CRC care continuum.

By identifying gaps in theoretical application, intervention specification, and reporting quality, this review highlights both the promise and the developmental stage of this emerging field. The findings offer oncology nurses and multidisciplinary teams a clear conceptual foundation for designing more rigorous, patient-centered behavioral interventions. Continued investment in well-designed, theory-driven trials and real-world implementation research will be essential to advance a scalable and sustainable science of behavioral support in colorectal cancer. Future BCW-informed CRC interventions should prioritize multi-level strategies that address opportunity-related barriers (*e.g.*, environmental restructuring and structured social support), which may help improve sustainability and transferability across settings.

## CRediT authorship contribution statement

**Siyu Lu:** Writing – original draft, Methodology, Formal analysis, Data curation, Conceptualization. **Qian Wu:** Supervision, Methodology, Conceptualization. **Lizhong Wang:** Supervision, Methodology, Conceptualization. **Xiaoli Liao:** Writing – review & editing, Supervision, Methodology, Conceptualization. **Dangheng Wei:** Writing – review & editing, Supervision, Methodology, Conceptualization. All authors have read and approved the final manuscript.

## Ethics statement

Not required.

## Data availability statement

The data that support the findings of this study are available from the corresponding author, XL, upon reasonable request.

## Declaration of generative AI and AI-assisted technologies in the writing process

During the preparation of this work, the authors used an AI-assisted tool to support language editing and improve clarity. After using this tool, the authors reviewed and edited the content as needed and take full responsibility for the content of the publication.

## Funding

This study received no external funding.

## Declaration of competing interest

The authors declare no conflict of interest.
