# Supplementary material for: Application of the behavior change wheel in supporting self-management among colorectal cancer survivors: A scoping review
Source: Asia Pac J Oncol Nurs. 2026 Feb 7;13:100916. doi: 10.1016/j.apjon.2026.100916 (PMC12992964; doi:10.1016/j.apjon.2026.100916)
Supplement: Multimedia component 1 [file mmc1.docx]

**Supplementary Table S1. Results of electronic database queries**

**Supplementary Table S2. Summary Matrix of 10 Included Studies**

**Supplementary Table S3. Quality Assessment of Included Studies Using the Cochrane Risk of Bias Tool (RoB 1)**

**Supplementary Table S4. JBI Qualitative Research Appraisal of Included Studies**

**Supplementary Table S1. Results of electronic database queries**

| **Databases** | **Search strategy** | **Results** |
| --- | --- | --- |
| PubMed | ("Colorectal Neoplasms"[MeSH] OR colorectal cancer*[Title/Abstract] OR colorectal neoplasm*[Title/Abstract] OR colon cancer*[Title/Abstract] OR rectal cancer*[Title/Abstract] OR colorectal carcinoma*[Title/Abstract] OR intestinal neoplasm*[Title/Abstract] OR bowel cancer*[Title/Abstract])  AND  ("behavior change wheel"[Title/Abstract] OR "behaviour change wheel"[Title/Abstract] OR BCW[Title/Abstract] OR "COM-B"[Title/Abstract] OR "COM-B model"[Title/Abstract] OR "COM B model"[Title/Abstract])  AND  ("Self-Management"[MeSH] OR "Self Care"[MeSH] OR "Patient Compliance"[MeSH] OR self-management[Title/Abstract] OR self care[Title/Abstract] OR self-care[Title/Abstract] OR adherence[Title/Abstract] OR compliance[Title/Abstract] OR screening[Title/Abstract] OR colonoscopy[Title/Abstract] OR bowel preparation[Title/Abstract] OR ostomy[Title/Abstract] OR stoma[Title/Abstract] OR wound care[Title/Abstract] OR symptom monitoring[Title/Abstract] OR symptom management[Title/Abstract] OR lifestyle[Title/Abstract] OR diet*[Title/Abstract] OR physical activity[Title/Abstract] OR exercise[Title/Abstract] OR follow-up[Title/Abstract] OR survivorship[Title/Abstract])  AND  ("2011/04/23"[Date - Publication] : "2025/04/30"[Date - Publication]) | 13 |
| Web of Science | TS=(("colorectal cancer*" OR "colorectal neoplasm*" OR "colon cancer*" OR "rectal cancer*" OR "intestinal neoplasm*" OR "intestinal carcinoma*" OR "intestinal cancer*")  AND ("behavior change wheel" OR "behaviour change wheel" OR BCW OR "COM-B" OR "COM-B model")  AND (intervention* OR "nursing intervention*")) AND PY=(2011-2025)  Web of Science Core Collection; Topic field; Editions: SCI-EXPANDED, SSCI. | 11 |
| Google Scholar | (colorectal cancer OR colorectal carcinoma)  AND (behaviour change wheel OR behavior change wheel OR COM-B model OR BCW)  AND (self management OR self care OR self monitoring OR adherence)  AND (nurse-led intervention OR nursing intervention)  No quotation marks; limited to 2011–2025; search date: 30 Apr 2025. | 59 |
| CNKI | SU=(结直肠癌+大肠癌+肠癌)  AND SU=(行为改变轮理论+BCW理论+COM-B模型+COM-B+行为改变理论+行为干预理论+行为模式理论)  AND SU=(自我管理+自我照护+健康行为+行为改变+依从性+遵医行为+饮食管理+情绪调节+症状监测+运动行为+复诊依从性)  AND SU=(干预+教育+数字健康+线上管理+远程干预+个性化干预+健康促进+生活方式干预) | 4 |
| Wanfang | 主题:(结直肠癌 OR 结肠癌 OR 直肠癌 OR 大肠癌)  AND 主题:(行为改变轮 OR 行为改变轮理论 OR BCW OR COM-B)  AND 主题:(自我管理 OR 依从性 OR 服药依从性 OR 康复 OR 并发症)  AND (发表时间:[2011 TO 2025]) | 15 |

CNKI, Chinese National Knowledge Infrastructure; Wanfang, Wanfang Data.

**Supplementary Table S2. Summary Matrix of 10 Included Studies**

| **Study** | **Country** | **Design** | **Sample** | **Target Behaviour** | **COM-B Components** | **BCW Functions** | **BCTs (reported / inferred*)** | **Intervention Duration** | **Outcome Direction** | **Effect Size** |
| --- | --- | --- | --- | --- | --- | --- | --- | --- | --- | --- |
| Yan J et al., 2022 | China | RCT | CRC stoma patients (*n* = 96) | Daily stoma self-care | C-Psych; C-Phys; O-Soc; M-Ref; M-Aut | Edu; Train; Pers; Enb; Mod | Instruction; demonstration; feedback; social support*; action planning* | 3 mo post-discharge | Self-care ability ↑ | Not reported |
| Wang Y & Zhu GL, 2020 | China | RCT | Postoperative CRC stoma patients (*n* = 94) | Perioperative stoma self-management | C-Psych; C-Phys; O-Soc; M-Ref; M-Aut | Edu; Train; Pers; Enb; EnvR | Instruction; demonstration; feedback; action planning* | 1 d pre-op; 2 d post-op; 1 mo post-discharge | SMA ↑; KAP ↑ | Not reported |
| Kerrison RS et al., 2018 | UK | RCT | Adults aged 55 y (*n* = 1383) | Flexible sigmoidoscopy screening uptake | C-Psych; O-Soc; M-Ref; M-Aut | Edu; Pers; Enb; Mod; EnvR | Info on consequences; instruction; prompts/cues; action planning*; pros & cons* | 12–24 mo; 12 wk follow-up | Screening uptake ↑ | Not reported |
| Guo JY, 2019 | China | RCT | Permanent CRC stoma patients (*n* = 80) | Daily stoma self-care | C-Psych; C-Phys; O-Soc; M-Ref; M-Aut | Edu; Pers; Train; Mod; Enb; EnvR | Instruction; demonstration; feedback; social support; prompts/cues; graded tasks* | 3 mo (pre-op + post-discharge) | Self-care ↑; complications ↓ | Not reported |
| Ze Y, 2023 | China | RCT | High-risk adults (*n* = 200) | Colonoscopy attendance | C-Psych; C-Phys; O-Soc; M-Ref; M-Aut | Edu; Train; Pers; Enb; Mod; EnvR | Info; instruction; demonstration; behavioral practice; social support; prompts/cues; problem solving*; action planning* | 2 wk (Motivation wk + Capability wk) | HBMQ ↑; uptake ↑; anxiety ↓ | Not reported |
| Zhu T., 2023 | China | Quasi-experimental | CRC polypectomy patients (*n* = 201) | Colonoscopy follow-up adherence | C-Psych; C-Phys; O-Soc; O-Phys; M-Ref; M-Aut | Edu; Train; Pers; Enb; Mod; EnvR | Instruction; demonstration; feedback; social support; graded tasks*; prompts/cues; action planning | 1 mo (4 peri-op timepoints) | Adherence ↑; complications ↓ | Not reported |
| Christie-de-Jong et al., 2022 | UK | Qualitative | Muslim women eligible for CRC screening (*n* = 20) | CRC screening intention | C-Psych; O-Soc; M-Ref; M-Aut | Not applicable | Themes: credible source; reframing; social support; cultural alignment | Not applicable | Screening intention ↑ | Not applicable |
| Gadd et al., 2024 | Australia | Qualitative | Adults aged 50–75 y (*n* =15) | iFOBT completion | C-Psych; C-Phys; O-Soc; O-Phys; M-Ref; M-Aut | Not applicable | Themes: simplified materials; GP encouragement; social support | Not applicable | Screening intention ↑; uptake ↑ | Not applicable |
| Kotte et al., 2024 | Sweden | Qualitative | Cancer survivors (*n* = 24; mixed tumour types; CRC subgroup = 13.6%) | Post-treatment exercise participation | C-Psych; C-Phys; O-Soc; O-Phys; M-Ref; M-Aut | Not applicable | Themes: guidance; motivation; trainer & peer support | Not applicable | Exercise engagement ↑ | Not applicable |
| Wang XY et al., 2023 | China | Qualitative | Elderly CRC patients (*n* = 30) | Preoperative exercise adherence | C-Psych; C-Phys; O-Soc; M-Ref; M-Aut | Not applicable | Themes: tailored guidance; family/professional support | Not applicable | Prehabilitation adherence ↑ | Not applicable |

CRC, colorectal cancer; RCT, randomized controlled trial; Edu, education; Pers, persuasion; Train, training; Enb, enablement; Mod, modelling; EnvR, environmental restructuring; C-Psych, psychological capability; C-Phys, physical capability; O-Soc, social opportunity; O-Phys, physical opportunity; M-Ref, reflective motivation; M-Aut, automatic motivation; BCTs, Behavior Change Techniques.

Effect sizes were not reported in any quantitative study. Items marked with * indicate inferred BCTs not explicitly reported by study authors. Intervention duration derives directly from original study methods and has not been altered. Qualitative studies did not include behavioral interventions; therefore BCW functions, BCTs, and duration are marked as not applicable. This table supports cross-study comparison and heterogeneity interpretation.

**Supplementary Table S3. Quality Assessment of Included Studies Using the Cochrane Risk of Bias Tool (RoB 1)**

| **Study** | **Random sequence generation** | **Allocation concealment** | **Blinding of participants and personnel** | **Blinding of outcome assessment** | **Incomplete outcome data** | **Selective reporting** | **Other bias** | **Overall risk** |
| --- | --- | --- | --- | --- | --- | --- | --- | --- |
| Yan J, Zhou J, & Zhang J, 2022 (China) | L | U | H | U | L | L | L | H |
| Wang Y, Zhu GL, 2020 (China) | H | U | H | U | L | L | H | H |
| Kerrison RS et al., 2018 (UK) | L | U | H | L | L | L | L | H |
| Guo JY, 2019 (China) | L | L | H | L | L | L | L | H |
| Ze Y, 2023 (China) | L | U | U | L | L | L | H | H |
| Zhu T., 2023 (China) | H | H | H | U | U | U | H | H |

L, Low risk; H, High risk; U, Unclear.

Risk of bias was assessed using the original Cochrane Risk of Bias tool (RoB 1). Domains included random sequence generation, allocation concealment, blinding of participants and personnel, blinding of outcome assessment, incomplete outcome data, selective reporting, and other potential sources of bias.

**Reference:** Higgins JPT, Altman DG, Gøtzsche PC, et al. The Cochrane Collaboration’s tool for assessing risk of bias in randomized trials. BMJ. 2011;343:d5928. doi:10.1136/bmj.d5928.

**Supplementary Table S4. JBI Qualitative Research Appraisal of Included Studies**

| JBI Evaluation Items (2020) | Christie-de Jong et al., 2022 (UK) | Gadd et al., 2024 (Australia) | Kotte et al., 2024 (Sweden) | Wang XY, Zhu XP, & Wu Q, 2023 (China) |
| --- | --- | --- | --- | --- |
| 1. Is there congruity between the stated philosophical perspective and the research methodology? | Y | Y | Y | Y |
| 2. Is there congruity between the research methodology and the research question or objectives? | Y | Y | Y | Y |
| 3. Is there congruity between the research methodology and the methods used to collect data? | Y | Y | Y | Y |
| 4. Is there congruity between the research methodology and the representation and analysis of data? | Y | Y | Y | Y |
| 5. Is there congruity between the research methodology and the interpretation of results? | Y | Y | Y | Y |
| 6. Is there a statement locating the researcher culturally or theoretically? | Y | N | Y | N |
| 7. Is the influence of the researcher on the research, and vice-versa, addressed? | N | Y | Y | U |
| 8. Are participants, and their voices, adequately represented? | Y | Y | Y | Y |
| 9. Is the research ethical according to current criteria, and is there evidence of ethical approval by an appropriate body? | Y | Y | Y | Y |
| 10. Do the conclusions drawn in the research report flow from the analysis or interpretation of the data? | Y | Y | Y | Y |
| Compliance rate (Y/10) | 9/10 | 9/10 | 10/10 | 8/10 |

Y, criterion met; N, criterion not met; U, unclear or insufficient information.

**Reference:** Joanna Briggs Institute. Critical Appraisal Tools. JBI. Accessed December 29, 2025. [https://jbi.global/critical-appraisal-tools](https://jbi.global/critical-appraisal-tools?utm_source=chatgpt.com)
