# Supplementary material for: Application of the behavior change wheel in supporting self-management among colorectal cancer survivors: A scoping review
Source: Asia Pac J Oncol Nurs. 2026 Feb 7;13:100916. doi: 10.1016/j.apjon.2026.100916 (PMC12992964; doi:10.1016/j.apjon.2026.100916)
Supplement: Multimedia component 2 [file mmc2.docx]

**Box 1. Representative PubMed search strategy**

(PubMed; searched 30 April 2025; publication date range: 23 April 2011–30 April 2025)

| ("Colorectal Neoplasms"[MeSH] OR colorectal cancer*[Title/Abstract] OR colorectal neoplasm*[Title/Abstract] OR colon cancer*[Title/Abstract] OR rectal cancer*[Title/Abstract] OR colorectal carcinoma*[Title/Abstract] OR intestinal neoplasm*[Title/Abstract] OR bowel cancer*[Title/Abstract])  AND  ("behavior change wheel"[Title/Abstract] OR "behaviour change wheel"[Title/Abstract] OR BCW[Title/Abstract] OR "COM-B"[Title/Abstract] OR "COM-B model"[Title/Abstract] OR "COM B model"[Title/Abstract])  AND  ("Self-Management"[MeSH] OR "Self Care"[MeSH] OR "Patient Compliance"[MeSH] OR self-management[Title/Abstract] OR self care[Title/Abstract] OR self-care[Title/Abstract] OR adherence[Title/Abstract] OR compliance[Title/Abstract] OR screening[Title/Abstract] OR colonoscopy[Title/Abstract] OR bowel preparation[Title/Abstract] OR ostomy[Title/Abstract] OR stoma[Title/Abstract] OR wound care[Title/Abstract] OR symptom monitoring[Title/Abstract] OR symptom management[Title/Abstract] OR lifestyle[Title/Abstract] OR diet*[Title/Abstract] OR physical activity[Title/Abstract] OR exercise[Title/Abstract] OR follow-up[Title/Abstract] OR survivorship[Title/Abstract])  AND  ("2011/04/23"[Date - Publication] : "2025/04/30"[Date - Publication]) |
| --- |
